# *Bifidobacterium bifidum* PRL2010 alleviates intestinal ischemia/reperfusion injury

**DOI:** 10.1371/journal.pone.0202670

**Published:** 2018-08-30

**Authors:** Sabrina Duranti, Valentina Vivo, Irene Zini, Christian Milani, Marta Mangifesta, Rosaria Anzalone, Leonardo Mancabelli, Alice Viappiani, Anna Maria Cantoni, Elisabetta Barocelli, Douwe van Sinderen, Simona Bertoni, Francesca Turroni

**Affiliations:** 1 Laboratory of Probiogenomics, Department of Chemical Sciences, Life Sciences, and Environmental Sustainability, University of Parma, Parma, Italy; 2 Food and Drug Department, University of Parma, Parma, Italy; 3 GenProbio srl, Parma, Italy; 4 Department of Veterinary Sciences, University of Parma, Parma, Italy; 5 APC Microbiome Institute and School of Microbiology, Bioscience Institute, National University of Ireland, Cork, Ireland; 6 Microbiome Research Hub, University of Parma, Parma, Italy; Universidade de Sao Paulo, BRAZIL

## Abstract

Mesenteric ischemia/reperfusion is a clinical emergency with high morbidity and mortality due to the transient reduction of blood supply to the bowel. In recent years, the critical contribution of gut microbiome to human health and proper gastrointestinal functions has gradually emerged. In the current study, we investigated the protective effects of five days supplementation with *Bifidobacterium bifidum* PRL2010 in a murine model of gut ischemia/reperfusion. Our findings indicate that animals pretreated with *B*. *bifidum* PRL2010 showed lower neutrophil recruitment in the lungs, remarkably reduced bacterial translocation and decreased transcription levels of TNFalpha and IL-10 both in liver and kidneys, at the same time increasing those of IL-12 in kidneys. Inhibiting the adhesion of pathogenic bacteria and boosting host innate immunity responses are among the possible protective mechanisms enacted by the probiotic. These results demonstrate that short-period treatment with *B*. *bifidum* PRL2010 is a potential strategy to dampen remote organ injury due to mesenteric ischemia/reperfusion.

## 1. Introduction

Mesenteric ischemia/reperfusion (I/R) is a life-threatening clinical emergency associated with different pathological conditions and surgical procedures, such as organ transplantation, bowel strangulation, vascular surgery and shock [1]. Gut epithelial cells are highly sensitive to I/R: the poor oxygenation, which results from the transient interruption of blood supply to the bowel, followed by reperfusion triggers an intense inflammatory response that typically causes progressive distal organ impairment and, consequently, a high rate of morbidity and mortality [2]. Several studies have shown that the most important consequences of mesenteric I/R injury are the loss of intestinal mucosal barrier function and subsequent translocation of bacteria and endotoxins from the gut into the blood and to distant organs [3, 4].

The human gut microbiota is a highly complex biological ecosystem that plays important roles in host health, such as immune stimulation, gut-mediated metabolism of essential nutrients, exclusion of gut pathogens and maintenance of appropriate mucosal function and intestinal barrier [5, 6]. In fact, a negative disturbance of gut microbiota composition, also known as dysbiosis, may impact on human health and may cause several gastrointestinal diseases [7–9].

Accordingly, probiotics, live non-pathogenic microorganism supplements, thanks to their documented ability to modulate the enteric microflora and to confer beneficial effects to the host through the modulation of immune and inflammatory responses [10, 11], have been proposed for the management of several intestinal disorders.

Bifidobacteria are dominant bacterial components of the gut microbiota in mammals, including humans during infancy. They therefore represent a valuable microbial prototype to investigate gut microbiota development, including factors that influence microbial establishment and persistence in the gut [12]. Furthermore, bifidobacteria have been evaluated in the prevention and treatment of different gastrointestinal infections such as *Clostridium difficile* infection as well as of intestinal disorders such as ulcerative colitis and Crohn’s disease [13].

In this context, we decided to focus our attention on *Bifidobacterium bifidum* PRL2010 (*B*. *bifidum* PRL2010), isolated from three-months old infants, that represents a strain very well described phenotypically and genotypically [14–16]. In previous *in vitro* and animal studies we have shown that *B*. *bifidum* PRL2010 is able to (i) adhere to the intestinal mucosa, (ii) prevent enteric pathogen colonization, and (iii) maintain gut homeostasis by down-regulating expression of chemokines and heat shock proteins and up-regulating defensins and tight junction proteins in the murine host [14–16].

Starting from these premises, the aim of the current study was to evaluate the effects of oral pre-treatment with *B*. *bifidum* PRL2010 on the local and systemic inflammatory responses triggered by mesenteric I/R in mice.

## 2. Materials and methods

### 2.1 Animals

Female adult Swiss mice, weighing approximately 18–25 g (Charles River, Italy), were kept under standard laboratory conditions with a controlled 12 hours light/dark cycle in a temperature-controlled room (22°C) with free access to water and standard chow. Mice were fasted 12 h before the experiment with free access to water. This investigation conformed to rules for the care and use of laboratory animals of the European Community and was in accordance with Italian Law (DLGS 26/2014). All procedures were approved by the University of Parma, and by the Italian Ministry of Health as executed by 99–2014 the Institutional Animal Care and Use Committee (Dipartimento per la Sanità Pubblica Veterinaria, la Nutrizione e la Sicurezza degli Alimenti Direzione Generale della Sanità Animale e del Farmaco Veterinario) and all efforts were made to minimize suffering.

### 2.2 Experimental protocol and ischemia/reperfusion (I/R) surgical procedure

Mice were administered for five days with *B*. *bifidum* PRL2010 10^9^ cells per day, prepared as previously described [17]. They were randomly divided into the following two groups: i) mice subjected to mesenteric I/R and ii) sham operated (SO) mice. Corresponding control animals were represented by I/R or SO mice supplemented with vehicle (glucose 5%).

After 12h fasting with free access to water, mice were anaesthetized with pentobarbital (70 mg/kg i.p.) and, after a midline laparotomy, the small bowel was displaced to the left side. The superior mesenteric artery (SMA) was identified and temporarily occluded with a microvascular clip. After 45 minutes of ischemia, during which the intestine was kept moisturized through a wet cotton gauze, the clamp was removed and reperfusion was allowed. The midline incision of the abdominal wall was then closed by two-layer sutures. Sham operated (SO) mice were subjected to the same surgical procedure and intestinal manipulation without occlusion of the SMA. After five hours of reperfusion, animals were euthanized through CO_2_ inhalation and distal small intestine (jejunum and ileum), lung, liver, kidneys, spleen and blood were collected and processed for histological, biochemical or quantitative-real time PCR analysis.

### 2.3 Bacterial strains and culture conditions

*B*. *bifidum* PRL2010 cultures were incubated in anaerobic atmosphere (2.99% H_2_, 17.01% CO_2_ and 80% N_2_) in a chamber (Concept 400, Ruskin) in the Man-Rogosa-Sharp (MRS) (Scharlau Chemie, Barcelona, Spain) supplemented with 0.05% (w/v) L-cysteine hydrochloride and incubated at 37°C for 16 h.

### 2.4 Murine exposure to bifidobacteria

Each animal was trained to spontaneously drink 100 μl of vehicle (5% glucose) over less than 5 minutes from a syringe daily for one week. After training, mice were administered, in the same voluntary way, 100 μl of suspension containing approximately 10^9^ CFU cells of *B*. *bifidum* PRL2010 for five consecutive days. Bacterial inocula were prepared by growing bifidobacterial cells, anaerobically overnight at 37°C in MRS broth. Cultures were harvested by centrifugation (3000 rpm for 8 minutes), washed and resuspended in 100 μl of vehicle (5% glucose). The viable count of each inoculum was determined by retrospective plating on MRS. Control animals received during the same period only 100 μl liquid vehicle (i.e., 5% glucose solution without bacterial cells).

### 2.5 Detection of bifidobacteria by quantitative PCR (qPCR) in fecal samples

Evaluation of the presence of bifidobacteria in the murine fecal samples, collected before and after 1, 4 and 7 days from the beginning of supplementation, was performed by quantitative real-time qPCR using previously described genus-specific primers [18, 19]. In order to estimate the level of bifidobacterial transiently present in each animal, a qPCR approach using strain specific primers based on strain-specific genes were used on DNA extracted from fecal samples. Murine fecal samples were subjected to DNA extraction using the QIAampDNA Stool Mini kit following the manufacturer’s instructions (Qiagen Ltd., Strasse, Germany).

For *B*. *bifidum* PRL2010, primers Bbif_0282Fw (5’-GCGAACAATGATGGCACCTA-3’) and Bbif_0282Rv (5’-GTCGAACACCACGACGATGT-3’) were used, which target a unique specific sequence identified in the genome of strain PRL2010 [20].

qPCR reactions were performed on MicroAmp optical plates sealed with MicroAmp optical caps (Applied Biosystems, Foster City, CA) and amplifications were carried out in CFX96 (Biorad) using SYBR Green PCR Master Mix (Applied Biosystems). Thermal cycling consisted of an initial cycle of 95˚C for 10 min followed by 40 cycles of 95˚C for 15 sec and 60˚C for 1 min. DNA extracts from cultures of *B*. *bifidum* PRL2010 were used to obtain a standard curve. Samples were analyzed in duplicate in at least two independent PCR runs.

### 2.6 Bacterial translocation to distant organs

In order to evaluate bacterial translocation, animals were euthanized and blood, kidney, liver and spleen were removed and then used for DNA extraction. To estimate the overall levels of bacteria a qRT-PCR approach was used involving the eubacterial universal primers Probio_Rev (5’-ATTACCGCGGCTGCT-3’) and Probio_Uni (5’-CCTACGGGRSGCAGCAG-3’) employing qRT-PCR conditions as previously described [21].

### 2.7 Myeloperoxidase activity

Myeloperoxidase (MPO) activity, index of tissue neutrophil infiltration, was determined in intestinal and pulmonary tissues according to Krawisz’s modified method [22]. Briefly, intestinal tissue was homogenized (1:10, v v^−1^) in a solution containing aprotinin 1 μg ml^−1^ in 100 mM potassium phosphate buffer (pH 7.4) and centrifuged for 20 min at 7000 x g at 4°C. Pellets were re-homogenized (1:5, v v^−1^) in 50 mM potassium phosphate buffer (pH 6.0) containing 0.5% hexadecylthrimethyl-ammonium bromide and aprotinin 1 μg ml^−1^, subjected to three cycles of freezing (20 min -196°C) and thawing (20 min 37°C) and centrifuged for 30 min at 5000 x g at 4°C. 100 μl of the supernatant was mixed with 900 μl of a buffer solution containing o-dianisidine (0.167 mg ml^−1^) and 0.0005% H_2_O_2_. The rate of change in absorbance was measured spectrophotometrically at 470 nm. One unit of MPO was defined as the quantity of enzyme degrading 1 μmol of peroxide per minute at 25°C. Data were expressed in units/gram of dry weight tissue.

### 2.8 Malondialdehyde assay

The formation of malondialdehyde (MDA) was used as index of lipoperoxidation. Tissue MDA levels were determined following Ohkawa’s method [23]. Ileal samples were homogenized in 1.15% KCl solution (1:10, v v^−1^). 100 μl of the homogenate was added to a solution containing 200 μl of 8.1% sodium dodecyl sulphate, 1.5 ml of 20% acetic acid (pH 3.5), 1.5 ml of 0.8% thiobarbituric acid and 700 μl distilled water. Samples were then boiled for 1 h at 95°C and centrifuged at 3000×g for 10 min. The absorbance of the supernatant was measured by spectrophotometry at 532 nm. Data were expressed in nmol/gram of dry weight tissue.

### 2.9 Vascular permeability

To assess interstitial edema formation, index of increased capillary permeability of intestinal mucosa, intestinal samples were removed and opened along the anti-mesenteric border and wet weight was recorded. After 24 h at room temperature the samples were then weighed to obtain the dry weight. Tissue water content was calculated using the wet-to-dry weight ratio according to Moore-Olufemi: (wet weight − dry weight)/dry weight [24].

### 2.10 RNA isolation, reverse transcription and gene expression

Total RNA was isolated following a previously described protocol from liver and kidneys [21]. Reverse transcription to cDNA was performed with iScript Select cDNA synthesis kit (Bio-Rad Laboratories) using the following thermal cycle: 5 min at 25°C, 30 min at 45°C and 8 min at 85°C. The mRNA expression levels of cytokines were analyzed with SYBR green technology in quantitative Real-Time PCR (Biorad) on a Bio-RAD CFX96 system. Gene expression was normalized relative to a housekeeping gene as previously described [25].

### 2.11 Intestinal and lung histology

Samples of intestine and lungs were collected from the different groups of animals to determine the level of histological injury. After euthanasia, the trachea was cannulated with PE-50 tubing and the lungs were fixed in situ via the cannula with 10% neutral buffered formalin, before the thorax was opened. Intestinal tissues were excised, flushed with saline and immersion-fixed in 10% neutral buffered formalin overnight. Fixed tissues were then dehydrated and embedded in paraffin. Transverse 5-μm sections were cut, stained with hematoxylin-eosin and examined in a light microscope (Nikon Eclipse E800) by a person unaware of the treatment. For each animal, at least five sections were cut from the distal portion of small intestine (jejunum-ileum) and from lungs. Intestinal mucosal damage was graded according to Chiu’s score [26] (0, normal mucosa; 1, development of sub-epithelial Gruenhagen’s space; 2, lifting of epithelial layer from the lamina propria; 3, massive epithelial lifting; 4, denuded villi; 5, digestion and disintegration of lamina propria). The degree of lung injury was determined by applying the scoring system described by Matute-Bello et al. [27] that graded congestion of alveolar septae (0: septae thin and delicate; 1: congested alveolar septae in 1/3-2/3 of the field; 3: congested alveolar septae in >2/3 of the field), intra-alveolar cell infiltrates (0:<5 intra-alveolar cells per field; 1: 5 to 10 intra-alveolar cells per field; 2: 10 to 20 intra-alveolar cells per field; 3: >20 intra-alveolar cells per field) and alveolar hemorrhage (0: no hemorrhage; 1: at least 5 erythrocytes per alveolus in 1 to 5 alveoli; 2: at least 5 erythrocytes per alveolus in 5 to 10 alveoli; 3: at least 5 erythrocytes per alveolus in >10 alveoli). Lung injury score was produced by the sum of the three independent variables.

The average value of histological score determined for the small intestine and for the lungs of each animal was pooled with those determined for the other animals of the same experimental group and the median value was determined. Quantitative analyses of the height of intestinal villi (V) and depth of intestinal crypts (C) were performed using ImageJ software following the method described by Pires et al. [28]. The mean villus height, crypt depth and villus/crypt ratio were calculated for each animal and pooled with those determined for other animals of the same experimental group.

### 2.12 Goblet cells analysis

Hematoxylin-eosin stained sections of intestinal tissues were stained for mucin-containing Goblet cells and mucous production using periodic acid-Schiff (PAS). The number of Goblet cells was counted in each section: the average number of Goblet cells per section was determined for each animal and then pooled with those determined for the other animals of the same experimental group and the mean value was calculated.

### 2.13 Source of drugs and chemicals

Myeloperoxidase was purchased from Calbiochem (Darmstadt, Germany). Sodium dodecyl sulphate and aprotinin were purchased from PanReac AppliChem ITW Reagents (Darmstadt, Germany). KCl and acetic acid were purchased from Analar Normapur, VWR (Dublin, Ireland). All other chemicals of reagent grade were obtained from Sigma Aldrich (St. Louis, MO, USA).

### 2.13 Statistical analysis

Data are expressed as means ± standard error (SE) unless otherwise specified. Comparison among groups was made using two-way or one-way analysis of variance (ANOVA) followed respectively by Bonferroni’s or Dunn’s post-test (P<0.05, P<0.01 and P<0.001 indicate respectively statistically significant, highly significant and extremely significant differences). All analyses were performed using Prism 5 program (GraphPad Soft-ware Inc., San Diego, CA) on a Macintosh computer.

## 3. Results

### 3.1 *B*. *bifidum* PRL2010 administration attenuates intestinal ischemia/reperfusion (I/R) injury

In order to assess the activity of *B*. *bifidum* PRL2010 against intestinal I/R injury under *in vivo* conditions, mice were pretreated with 10^9^ CFU/day of *B*. *bifidum* PRL2010 for five consecutive days before transient occlusion of SMA. Data were compared with sham operated (SO) mice supplemented with *B*. *bifidum* PRL2010 following the same experimental procedures except for SMA occlusion. Furthermore, two control groups were included [i.e., vehicle-treated I/R and SO mice]. *B*. *bifidum* PRL2010 colonization was monitored by quantitative-real time PCR on fecal samples collected at various time points during the experiment (S1 Fig).

**Fig 1:**
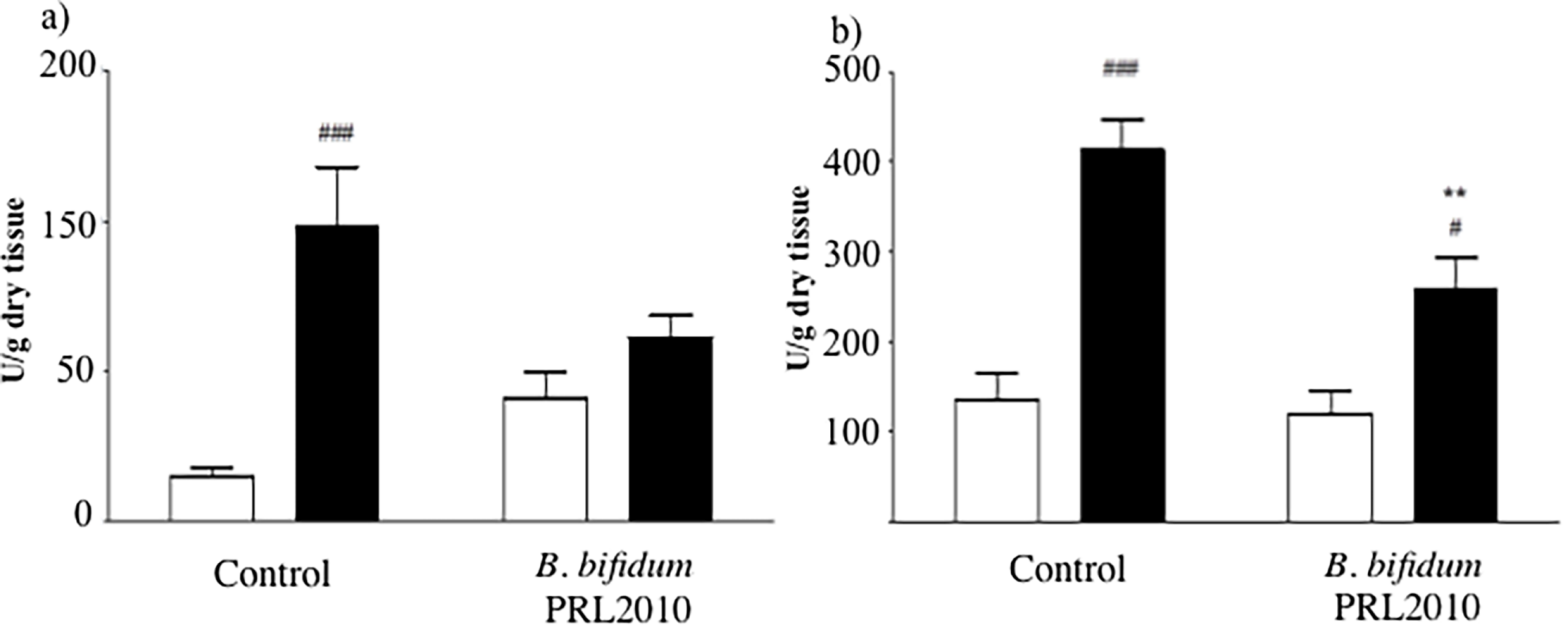
Effects of *B*. *bifidum* PRL2010 on myeloperoxidase activity in I/R mice. MPO activity, index of neutrophil infiltration, assessed in intestinal (a) and lung (b) tissue excised from SO (white columns) and I/R (black columns) mice orally administered with vehicle (Control), or supplemented with *B*. *bifidum* PRL2010 (n = 8–10 animals per group). Two-way ANOVA followed by Bonferroni’s post test: ^#^P<0.05; ^###^P<0.001 *vs*. corresponding SO mice; **P<0.01 *vs*. corresponding vehicle-treated group.

Notably, we found that in vehicle-treated mice, ischemic injury of 45 min followed by 5 h reperfusion significantly increased mortality (7 mice died out of 20 animals subjected to I/R) and augmented intestinal MPO activity, considered as an index of neutrophil accumulation, when compared with SO animals (P<0.001). Oral administration of *B*. *bifidum* PRL2010 did not improve survival of mice (8 mice died out of 23 animals subjected to I/R) but decreased neutrophil infiltration induced by I/R and slightly increased that triggered by simple intestinal manipulation in SO group, when compared to the respective vehicle-treated animals (Fig 1A). Furthermore, in lung tissues, MPO activity was significantly increased as a result of intestinal I/R injury when compared to MPO values obtained for SO mice (P<0.001). Notably, *B*. *bifidum* PRL2010 pre-treatment was shown to significantly decrease leukocyte recruitment in I/R mice when compared to the corresponding control group (P<0.01) (Fig 1B).

**Fig 2:**
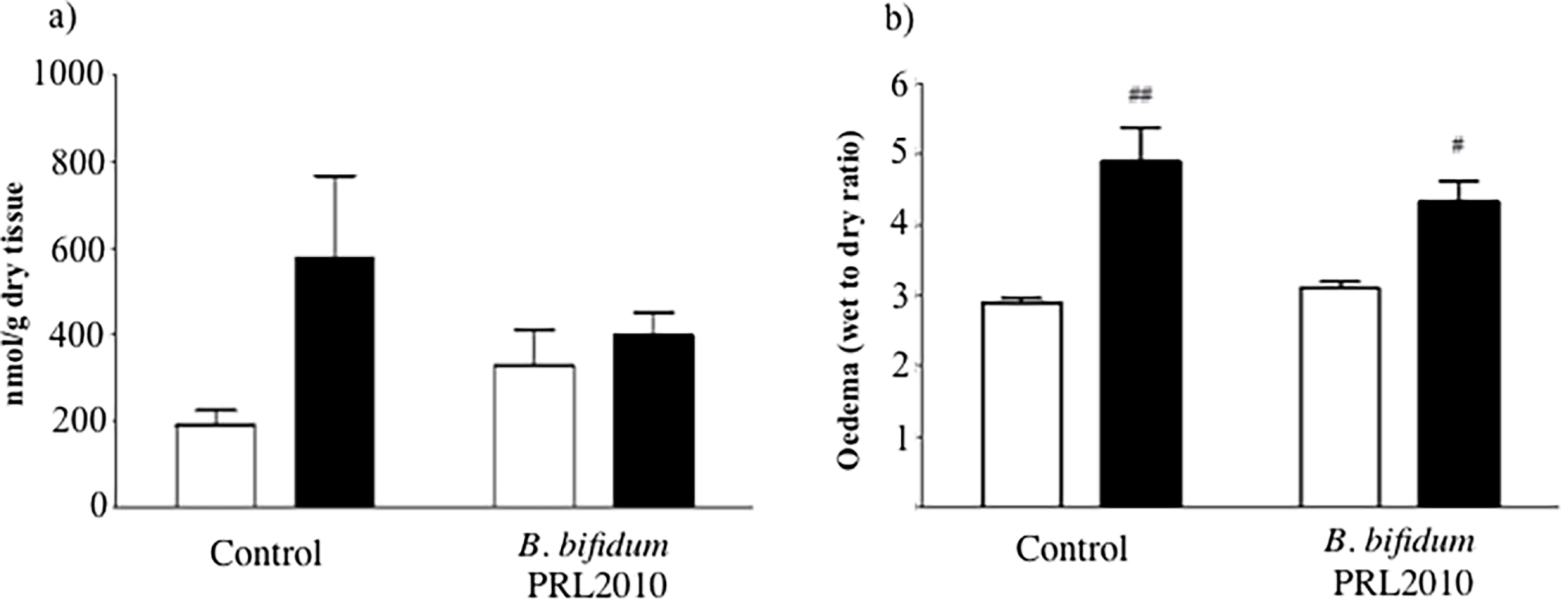
Effects of *B*. *bifidum* PRL2010 on oxidative stress and vascular permeability in I/R mice. MDA levels (a) and edema (b), expressed as wet-to-dry weight ratio, in intestinal tissues excised from SO (white columns) and I/R (black columns) mice orally administered with vehicle (Control) or supplemented with *B*. *bifidum* PRL2010 (n = 8–10 animals per group). Two-way ANOVA followed by Bonferroni’s post test: ^#^P<0.05; ^##^P<0.01 *vs*. corresponding SO mice.

Oxidative stress plays a key role in the pathogenesis of several clinical conditions, such as neurological disorder, diabetes, aging, and lung disease and, in particular, gastrointestinal disorders [29]. The transient occlusion of SMA augmented, although not significantly, intestinal tissue malondialdehyde levels, index of lipid peroxidation, compared to those of SO mice. Following *B*. *bifidum* PRL2010 treatment, comparable values of oxidative stress were observed in SO and I/R mice (Fig 2A).

Moreover, a consistent increase in gut vascular permeability was detected after I/R injury in the control group (P<0.01). Five-days administration of *B*. *bifidum* PRL2010 was ineffective in counteracting the I/R-induced plasma extravasation (Fig 2B).

### 3.2 Pretreatment with *B*. *bifidum* PRL2010 prevents bacterial translocation to distant organs

**Fig 3:**
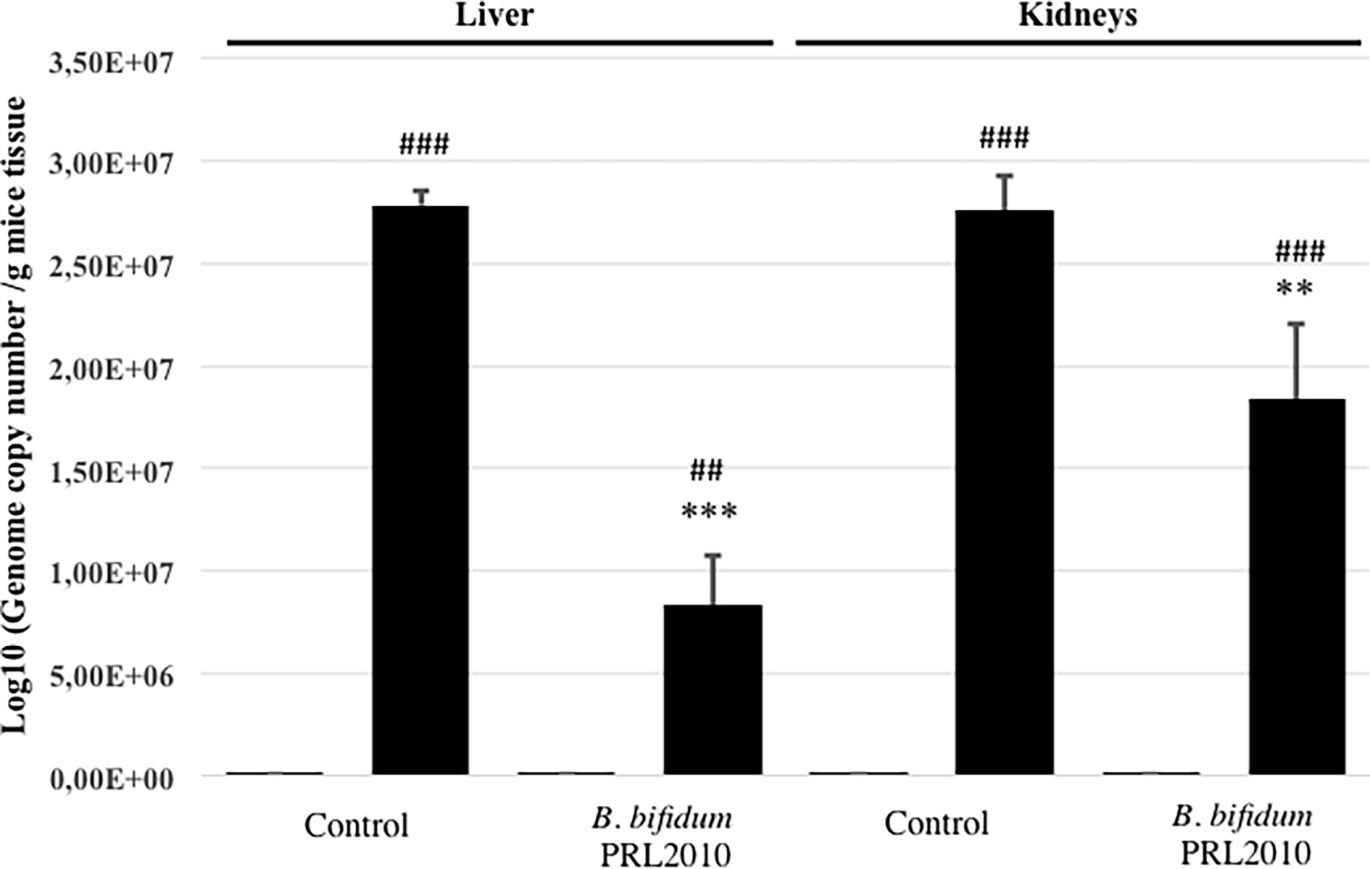
Effects of *B*. *bifidum* PRL2010 on bacterial translocation in I/R mice. qPCR analysis evaluated bacterial translocation in liver and kidneys samples excised from SO (white columns) and I/R (black columns) mice orally administered with vehicle (Control) or supplemented with *B*. *bifidum* PRL2010 (n = 5 animals per group). Each pillar represents the mean±SD of the log-transformed population size. Two-way ANOVA followed by Bonferroni’s post test: ^##^P<0.01, ^###^P<0.001 *vs*. corresponding SO mice; **P<0.01, ***P<0.001 *vs*. vehicle-treated I/R mice.

The loss of gut barrier function may cause bacterial translocation from the gut to peripheral organs [3]. In this context, we investigated bacterial translocation in several tissues collected from euthanized mice by means of qPCR. Interestingly, qPCR data highlighted that no bacteria could be detected in a numbers of organs and tissues assayed (e.g. blood, liver, kidney and spleen) excised from SO mice or in blood and spleen of I/R mice. Mesenteric I/R damage elicited a massive increase of bacterial translocation to peripheral organs such as liver and kidneys. However, oral administration of *B*. *bifidum* PRL2010 in I/R mice was shown to significantly reduce the bacterial translocation levels in both organs (P<0.01 for kidneys and P<0.001 for liver) (Fig 3).

### 3.3 Impact of *B*. *bifidum* PRL2010 on selected immune parameters

**Fig 4:**
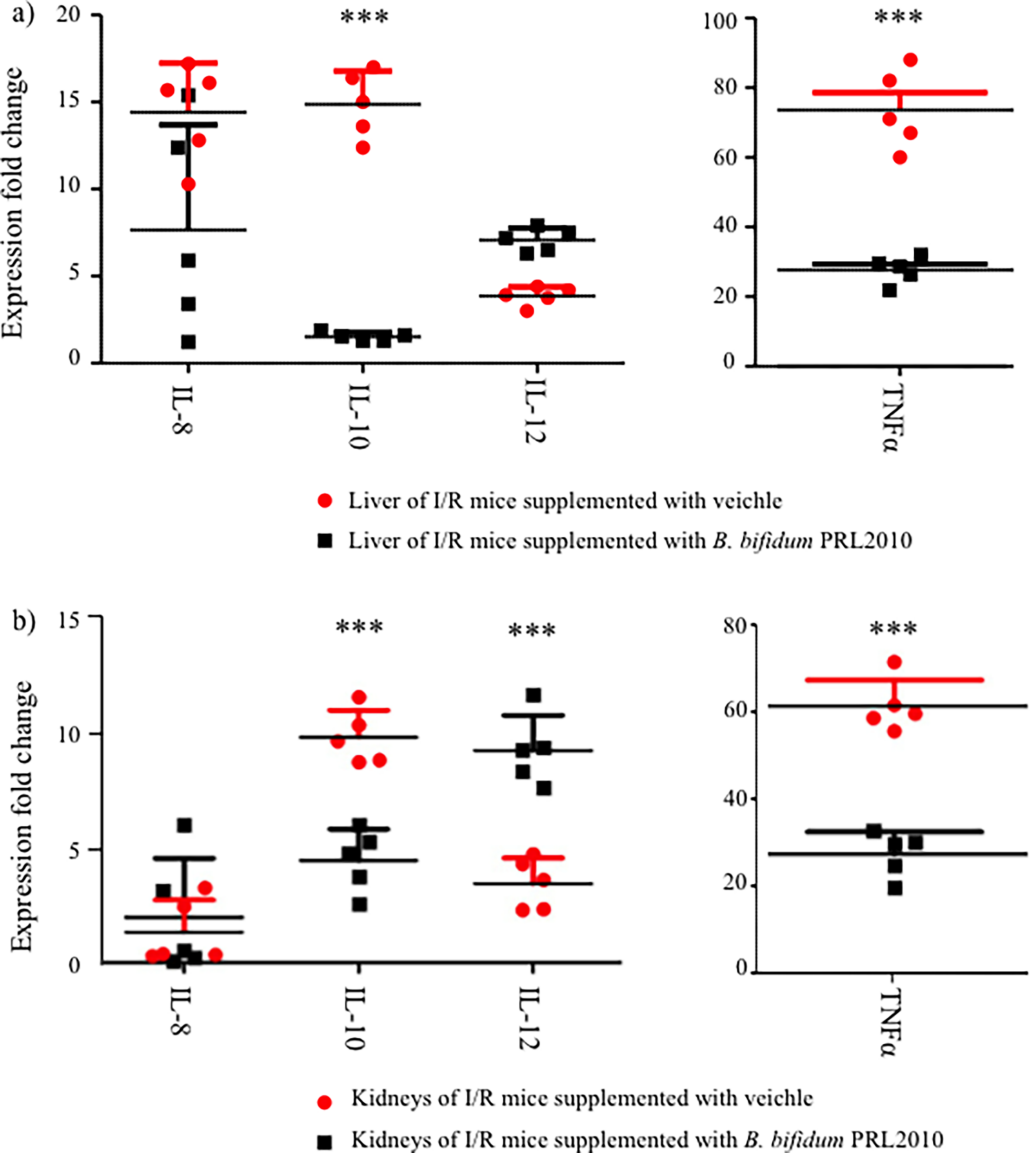
Effects of *B*. *bifidum* PRL2010 on the cytokine response in I/R mice. Panel a displays the relative transcription levels of IL-8, IL-10, IL-12 and TNFalpha in the liver of I/R mice supplemented with vehicle (designated with red circles) or with *B*. *bifidum* PRL2010 (designated with black squares). Panel b shows the relative transcription levels of the same cytokines in the kidneys of I/R mice supplemented with vehicle (red circles) or with *B*. *bifidum* PRL2010 (black squares). ***P<0.001 *vs*. vehicle-treated I/R mice, Student’s t-test. The *y*-axis represents the normalized expression level (ΔCt) according to CFX96 Bio-Rad software.

In order to explore the impact of *B*. *bifidum* PRL2010 on cytokines expression in organs such as liver and kidneys, we evaluated the induction of genes encoding for IL-10, IL-12, IL-8 and TNFalpha. In this context, we noticed different cytokine profile patterns between I/R mice supplemented with *B*. *bifidum* PRL2010 and I/R mice supplemented with the vehicle. Notably, tumor necrosis factor TNFalpha showed lower levels in liver and kidneys of mice subjected to intestinal I/R supplemented with *B*. *bifidum* PRL2010 with respect to mice subjected to intestinal I/R supplemented with vehicle. Furthermore, the supplementation of *B*. *bifidum* PRL2010 to I/R mice was shown to elicit a significant induction in kidneys of interleukin IL-12 and a lower interleukin IL-10 response both in the liver and in kidneys compared to vehicle-treated I/R mice (Fig 4).

The cytokine response of I/R mice treated with *B*. *bifidum* PRL2010 reinforced the notion that *B*. *bifidum* PRL2010 strain influences host innate immunity, an observation consistent with previous publications [14, 17].

### 3.4 *B*. *bifidum* PRL2010 and histology

**Fig 5:**
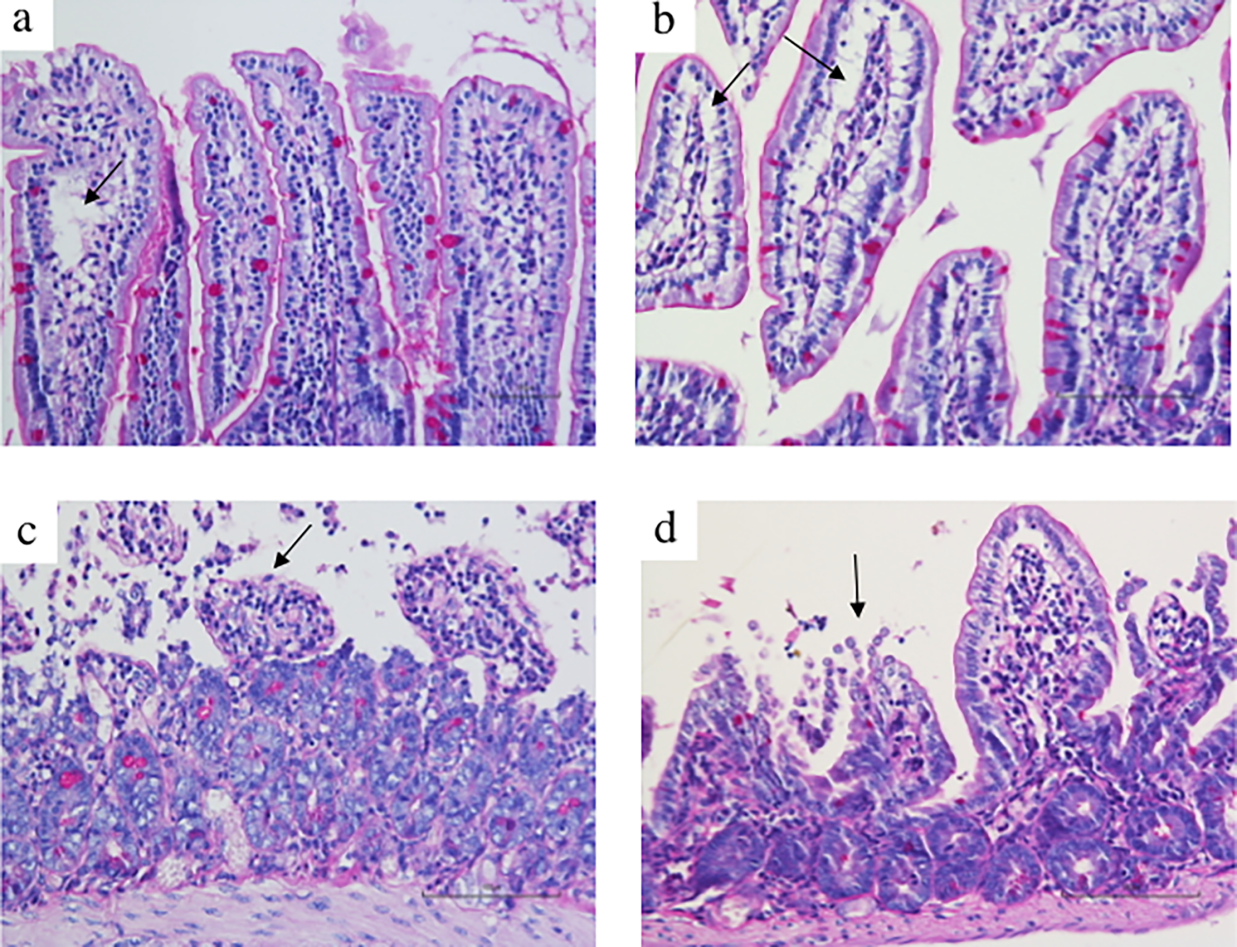
Intestinal histology. Representative micrographs of hematoxylin-eosin and PAS stained sections of jejunal-ileal specimens harvested from SO animals treated with vehicle (a) and *B*. *bifidum* PRL2010 (b) and from I/R mice pre-treated with vehicle (c) and *B*. *bifidum* PRL2010 (d) (scale bar: 100μm). Moderate expansion of Gruenhagen’s spaces (indicated by arrows in a and b) was visible both in vehicle- and in probiotic-treated SO mice. Mesenteric I/R caused extensive denudation of villi and, in some regions, digestion and disintegration of lamina propria (indicated by the arrow in c), with hemorrhages and ulceration; pre-treatment with *B*. *bifidum* PRL2010 was not able to prevent the I/R-induced mucosal lesions (indicated by the arrow in d).

**Fig 6:**
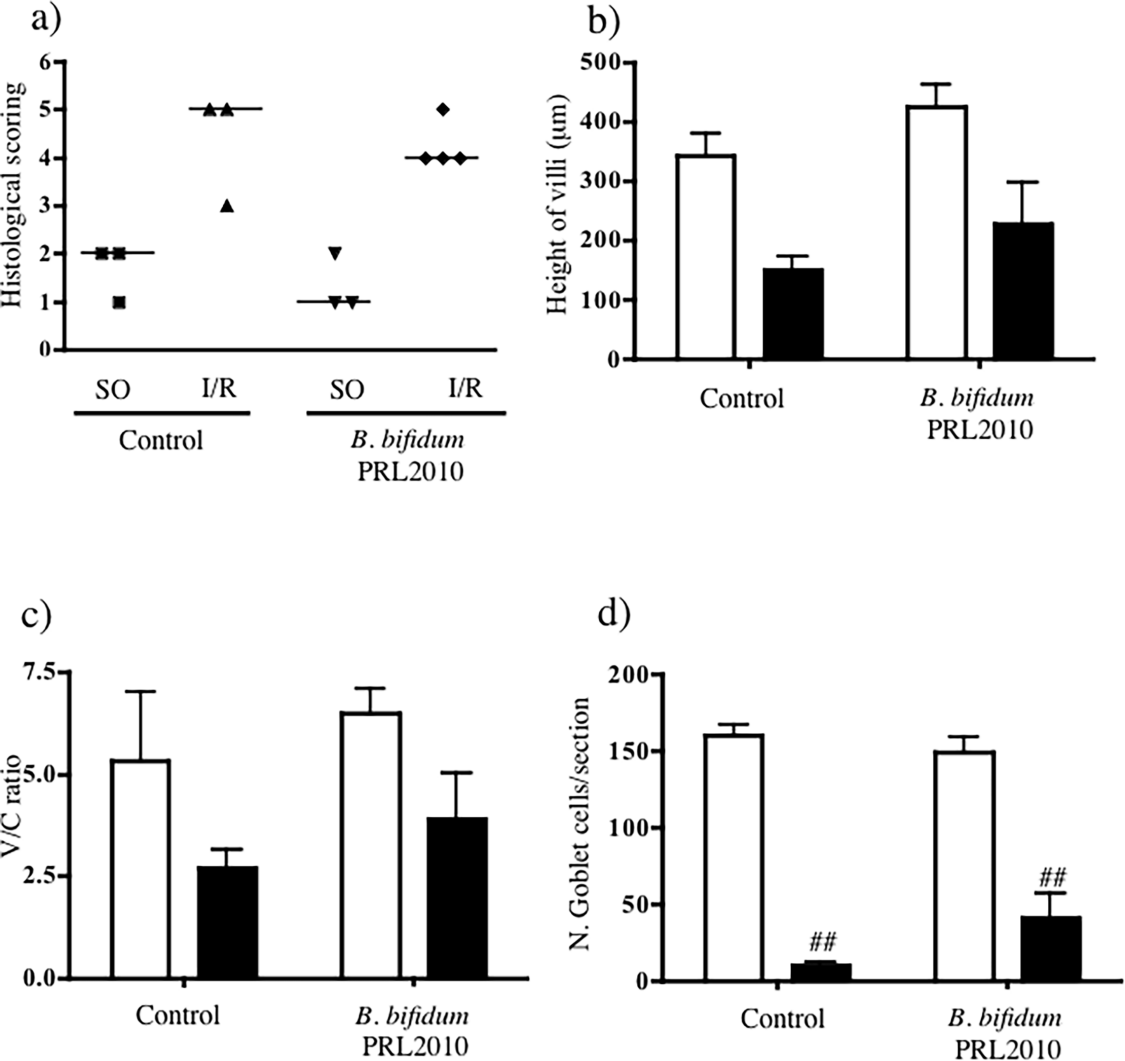
Intestinal morphometry. Panel a represents histological damage scoring of intestinal sections (horizontal bar at the median value) according to Chiu’s score [26]. Height of villi (μm) (b), V/C ratio (c) and number of Goblet cells (d) in intestinal tissues excised from SO (white columns) and I/R (black columns) mice orally administered with vehicle (Control) or supplemented with *B*. *bifidum* PRL2010 (n = 3–4 animals per group). Two-way ANOVA followed by Bonferroni’s post test: ^##^P<0.01 *vs*. corresponding SO mice.

Laparotomy and manipulation of the small intestine in SO mice produced a moderate expansion of the sub-epithelial space with lifting of the epithelial layer both in vehicle- (Fig 5A) and in *B*. *bifidum* PRL2010-treated (Fig 5B) mice. SMA occlusion for 45 minutes followed by 5h reperfusion caused a massive injury of intestinal mucosa with denudation of villi, digestion and disintegration of lamina propria, hemorrhage and ulceration, as shown by Fig 5C and by semi-quantitative scoring of gut injury (Fig 6A). Pre-treatment with *B*. *bifidum* PRL2010 was not effective in preserving mucosal integrity and in counteracting the remarkable I/R-induced intestinal damage (Figs 5D and 6A). The morphometric analysis confirmed the broad destruction of mucosal architecture provoked by mesenteric I/R and the inefficacy of *B*. *bifidum* PRL2010 in preventing it. Besides the difficulty in detecting villi to be measured after SMA occlusion, their height was markedly reduced compared with those in SO tissues (Fig 6B); similarly, the values of villi/crypts (V/C) ratios were curtailed in I/R tissues with respect to SO ones (Fig 6C). In both cases, the probiotic did not produce any significant improvement. As regards the number of Goblet cells, visualized by PAS staining, no changes were produced by supplementation with *B*. *bifidum* PRL2010 in SO mice; similarly, the dramatic reduction in their number, mainly ensuing from the mucosal degeneration caused by intestinal hypoxia, was only slightly attenuated by the short treatment with the probiotic (Fig 6D).

**Fig 7:**
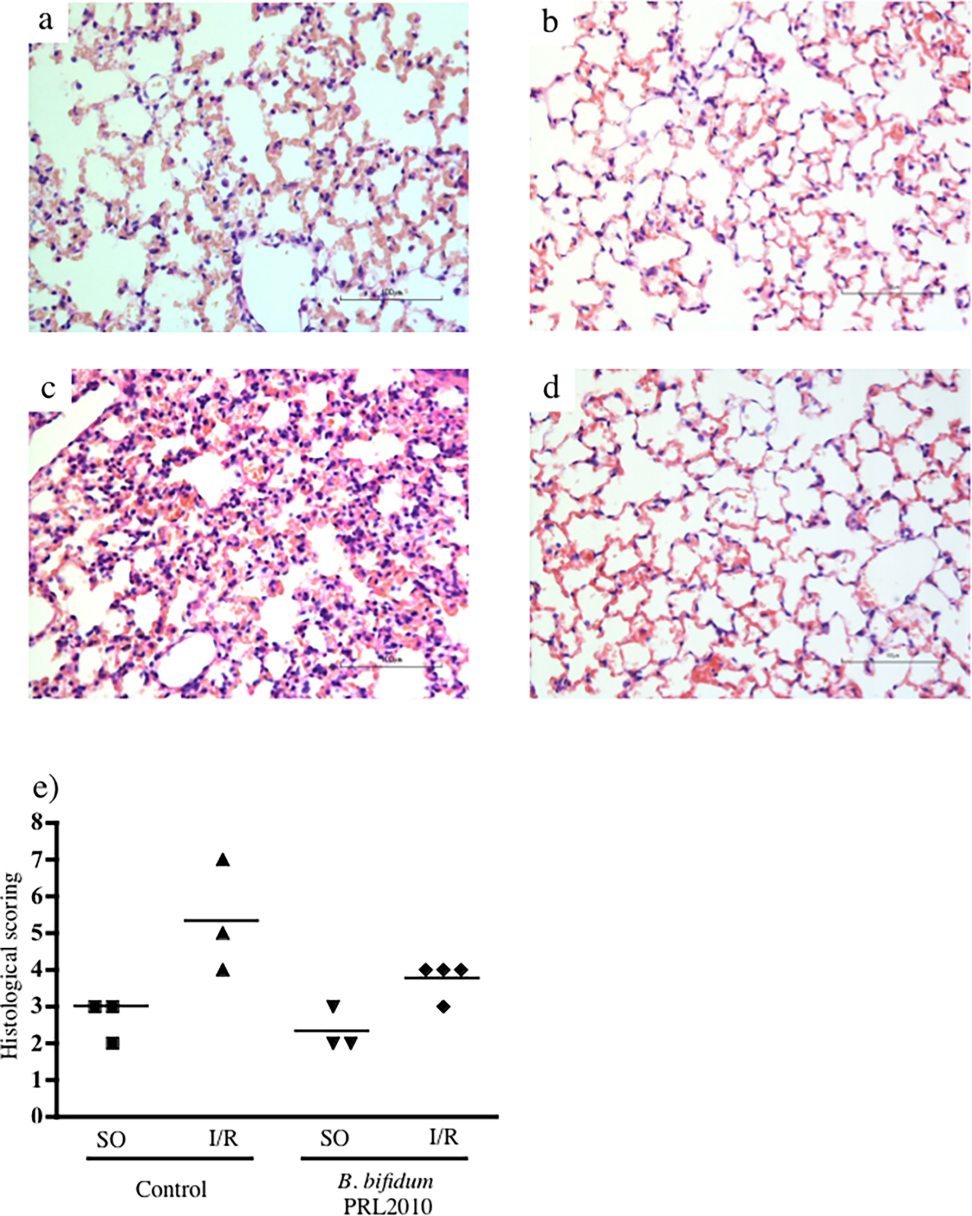
Lung histology. Representative hematoxylin-eosin stained sections of lung specimens harvested from SO animals treated with vehicle (a) and *B*. *bifidum* PRL2010 (b) and from I/R mice pre-treated with vehicle (c) and *B*. *bifidum* PRL2010 (d) (scale bar: 100μm). The acute lung injury caused by mesenteric I/R was characterized by diffuse congestion of septae, inter- and intra-alveolar cell infiltration and hemorrhage (c); pre-treatment with *B*. *bifidum* PRL2010 attenuated lung injury in all treated mice (d). Damage scoring of lung histological sections, according to Matute-Bello et al. [27], reported in panel e, shows the ability of *B*. *bifidum* PRL2010 to alleviate the I/R-induced remote injury (e).

As regards lung histological injury, laparotomy and small intestine manipulation caused only rare phenomena of septae congestion and very weak neutrophil infiltration, both in vehicle- and in *B*. *bifidum* PRL2010-treated rats (Fig 7A, 7B and 7E). Mesenteric I/R produced strong morphological changes in the lungs, inducing extensive congestion of septae, inter- and intra-alveolar infiltration of neutrophils and intra-alveolar hemorrhages (Fig 7C). Supplementation with *B*. *bifidum* PRL2010 attenuated lung injury: only moderate hyperemia of inter-alveolar capillaries and low-grade inter- and intra-alveolar infiltration of neutrophils and erythrocytes could be detected in I/R lungs (Fig 7D and 7E).

## 4. Discussion

In the present study, the transient occlusion of SMA triggered an extensive intestinal mucosal damage associated to an intense local and systemic inflammatory response: massive neutrophil infiltration in the gut and lung districts, marked increase of gut and lung vascular permeability and oxidative stress, remote bacterial translocation and TNFalpha and IL-10 up-regulation in liver and kidneys occurred. In these experimental conditions, we observed that a five-days treatment with *B*.*bifidum* PRL2010 was able to attenuate mesenteric I/R-induced changes: in fact, pre-treatment with probiotic dampened neutrophil infiltration, especially at the pulmonary level, moderately reduced oxidative stress and significantly decreased bacterial translocation and TNFalpha and IL-10 liver and kidneys transcription levels, while increasing those of IL-12 in kidneys. These findings are in line with Wang research, that recently demonstrated that 14 days oral pre-treatment with a pool of bifidobacteria was able to increase the expression of tight junctions proteins and to reduce bacterial translocation from gut to distant organs [30].

As concerns the evaluation of local inflammatory parameters, *B*. *bifidum* PRL2010 exerted a slight beneficial effect on neutrophils infiltration and lipid peroxidation in the gut after I/R injury, lowering MPO activity and driving MDA levels closer to the respective SO mice. *B*. *bifidum* PRL2010 previously showed immunomodulatory properties stimulating the host immune system during colonization and inducing different host genes expression. Among the main targeted genes, some chemokines and HSP encoding genes resulted down regulated, while defensin and tight junctions genes were up regulated [14, 17, 31]. On the basis of those immunomodulatory properties, the slight increase of neutrophil recruitment and lipid peroxidation observed in treated SO mice could be explained as a reinforcement of the innate system driven by the *B*. *bifidum* PRL2010, which might contribute to mitigate the degree of inflammation evoked by mesenteric I/R.

In our study, pre-treatment with *B*. *bifidum* PRL2010 did not affect intestinal plasma extravasation and did not preserve mucosal integrity, possibly due to the extensive damage triggered by hypoxia in our experimental model of mesenteric I/R. However, the remarkable reduction in leukocytes recruitment and the preservation of alveolocapillary membranes in the lungs, documented by biochemical and histological assays, along with the prevention of bacterial translocation and decrease of TNFalpha transcription levels in kidneys and liver highlight an undisputable protective action mediated by *B*. *bifidum* PRL2010. By now, it is well-known that, following transient mesenteric hypoxia, the loss of mucosal barrier integrity and the ensuing surge of luminal bacteria and toxins into the systemic circulation play a crucial role in the systemic inflammatory response syndrome (SIRS) and multi-organ dysfunction syndrome (MODS), primary causes of death after gut I/R [4]. Among different organs, lung is the most frequently compromised after mesenteric I/R injury, and respiratory failure is the first symptom of MODS. The favourable effects shown by *B*. *bifidum* PRL2010 in our experimental model testify therefore its ability to dampen the inflammatory response, confining it to the gut, and to prevent the involvement of distant organs in I/R-induced injuries.

The mechanism underlying this beneficial effect remains to be defined. Previous *in vitro* investigations demonstrated the ability of *B*. *bifidum* PRL2010 to reinforce epithelial barrier function through the stimulation of mucin secretion and by inhibiting the adhesion of pathogenic bacteria such as *E*. *coli* and *C*. *sakazakii* [15] whose overgrowth has been reported to play a key role in promoting bacterial translocation [32]. The reduced bacterial translocation as well as the decreased TNFalpha levels in liver and kidneys of I/R mice supplemented with *B*. *bifidum* PRL2010 compared to vehicle-treated I/R mice could likely result from the inhibition of pathogen bacteria adhesion to intestinal epithelium. On the other hand, less probable appears the change in the number of Goblet cells as potential protective factor put in place by *B*. *bifidum* PRL2010.

Another important aspect worth of consideration is the ability of *B*. *bifidum* PRL2010 to stimulate the transcription of IL-12 while decreasing that of IL-10 in kidneys: this effect, already documented in the cecal mucosa of Balb/c mice [14], could be interpreted as a further proof of the immunostimulatory properties of this strain of *B*. *bifidum*. Indeed, starting from this evidence, we can speculate that, following colonization, *B*. *bifidum* PRL2010 promotes innate immunity responses and the release of IL-12 by macrophages and dendritic cells, simultaneously dampening that of the immune-suppressive cytokine IL-10. The subsequent activation of adaptive Th1 cells would eventually contribute to prevent the dissemination of gut bacteria to remote organs. Similarly, lactobacilli, such as Lactobacillus casei Shirota or Lactobacillus reuteri ATCC 23272, proved to induce Th1 cells via the increased production of IL-12 generated by native immune cells [33].

In conclusion, our findings suggest that short-time treatment of mice, subjected to mesenteric I/R, with *B*. *bifidum* PRL2010 was significantly effective in reducing bacterial translocation and the subsequent systemic inflammatory response, despite its apparent lack of efficacy in protecting the intestinal barrier integrity. The treatment with this single strain could be therefore helpful to prevent distal organs impairment, an issue considered the primary cause of death from intestinal I/R injury: it is expected that longer periods of treatment provide additional benefits, eventually increasing also the rate of survival.

## Supporting information

S1 Fig Panel a shows a schematic representation of the design of the murine trial. Panel b displays population sizes of *B*. *bifidum* PRL2010 strain transiently present in the intestine of Swiss mice (n = 14). Each point represents the average of the log-transformed population size ± standard deviation. On x axis the time of feces collection is indicated with T0 = before *B*. *bifidum* PRL2010 administration, T1 = 1 day, T2 = 4 days and T3 = 7 days after the beginning of *B*. *bifidum* PRL2010 administration.
(TIFF)

## References

[1] Bertoni S, Ballabeni V, Barocelli E, Tognolini M. Mesenteric ischemia-reperfusion: an overview of preclinical drug strategies. Drug Discov Today 2018; 10.1016/j.drudis.2018.05.034 29857163

[2] Carver TW, Vora RS, Taneja A. Mesenteric ischemia. Crit Care Clin 2016; 32: 155–71. 10.1016/j.ccc.2015.11.001 27016159

[3] Carden DL, Granger DN. Pathophysiology of ischemia-reperfusion injury. J Pathol 2000; 190: 255–66. 10.1002/(SICI)1096-9896(200002)190:3<255::AID-PATH526>3.0.CO;2-6 10685060

[4] Clark JA, Coopersmith CM. Intestinal crosstalk: a new paradigm for understanding the gut as the “motor” of critical illness. Shock 2007; 28: 384–93. 10.1097/shk.0b013e31805569df 17577136 PMC2084394

[5] Berg RD. The indigenous gastrointestinal microflora. Trends Microbiol. 1996;4(11):430–5. 8950812 10.1016/0966-842x(96)10057-3

[6] Bäckhed F, Ley RE, Sonnenburg JL, Peterson DA, Gordon JI. Host-bacterial mutualism in the human intestine. Science 2005; 307: 1915–20. 10.1126/science.1104816 15790844

[7] Hollister EB, Gao C, Versalovic J. Compositional and functional features of the gastrointestinal microbiome and their effects on human health. Gastroenterology 2014; 146: 1149–58. 10.1053/j.gastro.2014.01.052 PMC4181834 24486050

[8] Rajilic-Stojanovic M, Biagi E, Heilig HG, Kajander K, Tims S, de Vos WM. Global and deep molecular analysis of microbiota signatures in fecal samples from patients with irritable bowel syndrome. Gastroenterology 2011; 141: 1792–801. 10.1053/j.gastro.2011.07.043 21820992

[9] Sobhani I, Tap J, Roudot-Thoraval F, Roperch JP, Letulle S, Langella P et al Microbial dysbiosis in colorectal cancer (CRC) patients. Plos One 2011; 6: e16393 10.1371/journal.pone.0016393 21297998 PMC3029306

[10] Guarner F, Khan AG, Garisch J, Eliakim R, Gangl A, Thomson A, et al World Gastroenterology Organisation Global Guidelines: probiotics and prebiotics October 2011. J Clin Gastroenterol. 2012; 46(6): 468–81. 10.1097/MCG.0b013e3182549092 22688142

[11] Isolauri E, Kirjavainen PV, Salminen S. Probiotics: a role in the treatment of intestinal infection and inflammation? Gut. 2002; 50: III54–9. 10.1136/gut.50.suppl_3.iii54 11953334 PMC1867676

[12] Milani C, Mangifesta M, Mancabelli L, Lugli GA, James K, Duranti S et al Unveiling bifidobacterial biogeography across the mammalian branch of the tree of life. ISME J 2017; 11: 2834–2847. 10.1038/ismej.2017.138 28837128 PMC5702741

[13] Fedorak RN, Feagan BG, Hotte N, Leddin D, Dieleman LA, Petrunia DM et al The probiotic VSL#3 has anti-inflammatory effects and could reduce endoscopic recurrence after surgery for Crohn’s disease. Clin Gastroenterol Hepatol 2015; 13: 928–35. 10.1016/j.cgh.2014.10.031 25460016

[14] Duranti S, Gaiani F, Mancabelli L, Milani C, Grandi A, Bolchi A et al Elucidating the gut microbiome of ulcerative colitis: bifidobacteria as novel microbial biomarkers. FEMS Microbiol Ecol 2016; 92 (12). Pii: fiw191. 10.1093/femsec/fiw191 27604252

[15] Serafini F, Strati F, Ruas-Madiedo P, Turroni F, Foroni E, Duranti S, et al Evaluation of adhesion properties and antibacterial activities of the infant gut commensal Bifidobacterium bifidum PRL2010. Anaerobe 2013; 21: 9–17. 10.1016/j.anaerobe.2013.03.003 23523946

[16] Turroni F, Taverniti V, Ruas-Madiedo P, Duranti S, Guglielmetti S, Lugli GA, et al Bifidobacterium bifidum PRL2010 modulates the host innate immune response. Appl Environ Microbiol. 2014; 80(2):730–40. 10.1128/AEM.03313-13 24242237 PMC3911076

[17] Turroni F, Milani C, Duranti S, Mancabelli L, Mangifesta M, Viappiani A et al Deciphering bifidobacterial-mediated metabolic interactions and their impact on gut microbiota by multi-omics approach. ISME J 2016; 10: 1656–68. 10.1038/ismej.2015.236 26859770 PMC4918443

[18] Gueimonde M, Tölkkö S, Korpimäki T, Salminen S. New real-time quantitative PCR procedure for quantification of bifidobacteria in human fecal samples. Appl Environ Microbiol 2004; 70: 4165–9. 10.1128/AEM.70.7.4165-4169.2004 15240297 PMC444799

[19] Matsuki T, Watanabe K, Fujimoto J, Miyamoto Y, Takada T, Matsumoto K, et al Development of 16S rRNA-gene-targeted group-specific primers for the detection and identification of predominant bacteria in human feces. Appl Environ Microbiol. 2002; 68: 5445–51. 10.1128/AEM.68.11.5445-5451.2002 12406736 PMC129894

[20] Turroni F, Serafini F, Foroni E, Duranti S, O’Connell Motherway, Taverniti V et al Role of sortase-dependent pili of Bifidobacterium bifidum PRL2010 in modulating bacterium-host interactions. Proc Natl Acad Sci 2013; 110: 11151–6. 10.1073/pnas.1303897110 23776216 PMC3703987

[21] Milani C, Hevia A, Foroni E, Duranti S, Turroni F, Lugli GA, et al Assessing the fecal microbiota: an optimized ion torrent 16S rRNA gene-based analysis protocol. PLoS One 2013; 8:e68739 10.1371/journal.pone.0068739 23869230 PMC3711900

[22] Krawisz JE, Sharon P, Stenson WF. Quantitative assay for acute intestinal inflammation based on myeloperoxidase activity. Assessment of inflammation in rat and hamster models. Gastroenterology 1984, 87: 1344–50. 6092199

[23] Ohkawa H, Ohishi N, Yagi K. Assay for lipid peroxides in animal tissues by thiobarbituric acid reaction. Anal Biochem. 1979, 95: 351–8. 36810 10.1016/0003-2697(79)90738-3

[24] Moore-Olufemi SD, Kozar RA, Moore FA, Sato N, Hassoun HT, Cox CS Jr, et al Ischemic preconditioning protects against gut dysfunction and mucosal injury after ischemia/reperfusion injury. Shock 2005, 23: 258–63. 15718925

[25] Turroni F, Foroni E, Montanini B, Viappiani A, Strati F, Duranti S et al Global genome transcription profiling of Bifidobacterium bifidum PRL010 under in vitro conditions and identification of reference genes for quantitative real-time PCR. Appl Environ Microbiol 2011; 77: 8578–87. 10.1128/AEM.06352-11 22003014 PMC3233107

[26] Chiu CJ, McArdle AH, Brown R, Scott HJ, Gurd FN: Intestinal mucosal lesion in low-flow states. I. A morphological, hemodynamic and metabolic reappraisal. Arch Surg. 1970; 101: 478–83. 5457245 10.1001/archsurg.1970.01340280030009

[27] Matute-Bello G, Winn RK, Jonas M, Chi EY, Martin TR, Liles WC. Fas (CD95) induces alveolar epithelial cell apoptosis in vivo: implications for acute pulmonary inflammation. Am J Pathol 2001; 158: 153–61. 10.1016/S0002-9440(10)63953-3 11141488 PMC1850249

[28] Pires AL, da Silveira TR, da Silva VD. Digital morphometric and stereological analysis of small intestinal mucosa in well-nourished and malnourished children with persistent diarrhea. J Pediatr 2003; 79: 329–36. 14513132

[29] Aviello G, Knaus UG. ROS in gastrointestinal inflammation: rescue or sabotage? Br J Pharmacol 2016; 174: 1704–18. 10.1111/bph.13428 26758851 PMC5446568

[30] Wang H, Zhang W, Zuo L, Zhu W, Wang B, Li Q, et al Bifidobacteria may be beneficial to intestinal microbiota and reduction of bacterial translocation in mice following ischaemia and reperfusion injury. Br J Nutr. 2013, 109: 1990–8. 10.1017/S0007114512004308 23122253

[31] Turroni F, Ventura M, Buttò LF, Duranti S, O’Toole PW, Motherway MO, et al Molecular dialogue between the human gut microbiota and the host: a Lactobacillus and Bifidobacterium perspective. Cell Mol Life Sci 2014; 71: 183–203. 10.1007/s00018-013-1318-0 23516017 PMC11113728

[32] Batt RM, Rutgers HC, Sancak AA. Enteric bacteria: friend or foe?. J Small Anim Pract 1996; 37: 261–7. 8805096 10.1111/j.1748-5827.1996.tb02376.x

[33] Mohamadzadeh M, Olson S, Kalina WV, Ruthel G, Demmin GL, Warfield KL, et al Lactobacilli activate human dendritic cells that skew T cells toward T helper 1 polarization. Proc Natl Acad Sci USA 2005; 102: 2880–5. 10.1073/pnas.0500098102 15710900 PMC549474

