# Supplementary figures and images for: Bifidobacterium bifidum PRL2010 alleviates intestinal ischemia/reperfusion injury
Source: PLoS One. 2018 Aug 30;13(8):e0202670. doi: 10.1371/journal.pone.0202670 (PMC6116995; doi:10.1371/journal.pone.0202670)

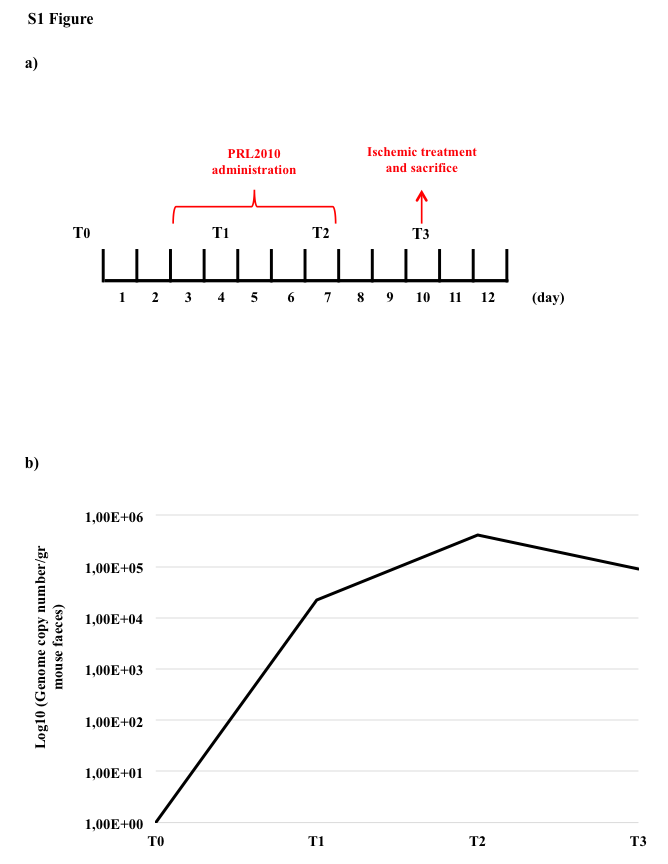

Supplement: S1 Fig — Panel b displays population sizes of B. bifidum PRL2010 strain transiently present in the intestine of Swiss mice (n = 14). Each point represents the average of the log-transformed population size ± standard deviation. On x axis the time of feces collection is indicated with T0 = before B. bifidum PRL2010 administration, T1 = 1 day, T2 = 4 days and T3 = 7 days after the beginning of B. bifidum PRL2010 administration. (TIFF) [file pone.0202670.s001.tiff]
